# Circulating miRNA panels as a novel non-invasive diagnostic, prognostic, and potential predictive biomarkers in non-small cell lung cancer (NSCLC)

**DOI:** 10.1038/s41416-024-02831-3

**Published:** 2024-08-30

**Authors:** Maryam Abdipourbozorgbaghi, Adrienne Vancura, Ramin Radpour, Simon Haefliger

**Affiliations:** 1 Department of Medical Oncology, Inselspital, Bern University Hospital, University of Bern, Bern, Switzerland; 2 Department of BioMedical Research (DBMR), University of Bern, Bern, Switzerland; 3 Graduate School of Cellular and Biomedical Sciences, University of Bern, Bern, Switzerland

**Keywords:** Prognostic markers, Diagnostic markers, Predictive markers

## Abstract

**Background:**

Non-small cell lung cancer (NSCLC) is characterised by its aggressiveness and poor prognosis. Early detection and accurate prediction of therapeutic responses remain critical for improving patient outcomes. In the present study, we investigated the potential of circulating microRNA (miRNA) as non-invasive biomarkers in patients with NSCLC.

**Methods:**

We quantified miRNA expression in plasma from 122 participants (78 NSCLC; 44 healthy controls). Bioinformatic tools were employed to identify miRNA panels for accurate NSCLC diagnosis. Validation was performed using an independent publicly available dataset of more than 4000 NSCLC patients. Next, we correlated miRNA expression with clinicopathological information to identify independent prognostic miRNAs and those predictive of anti-PD-1 treatment response.

**Results:**

We identified miRNA panels for lung adenocarcinoma (LUAD) and squamous cell carcinoma (LUSC) diagnosis. The LUAD panel consists of seven circulating miRNAs (miR-9-3p, miR-96-5p, miR-147b-3p, miR-196a-5p, miR-708-3p, miR-708-5p, miR-4652-5p), while the LUSC panel comprises nine miRNAs (miR-130b-3p, miR-269-3p, miR-301a-5p, miR-301b-5p, miR-744-3p, miR-760, miR-767-5p, miR-4652-5p, miR-6499-3p). Additionally, miR-135b-5p, miR-196a-5p, miR-31-5p (LUAD), and miR-205 (LUSC) serve as independent prognostic markers for survival. Furthermore, two miRNA clusters, namely miR-183/96/182 and miR-767/105, exhibit predictive potential in anti-PD-1-treated LUAD patients.

**Conclusions:**

Circulating miRNA signatures demonstrate diagnostic and prognostic value for NSCLC and may guide treatment decisions in clinical practice.

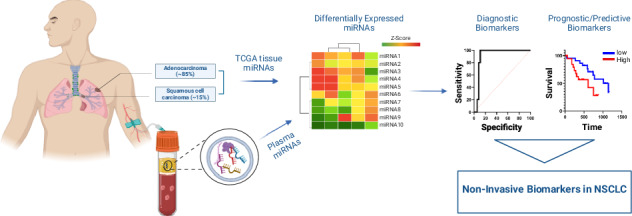

## Background

Lung cancer is the leading cause of cancer-related death worldwide [1]. The two most common types of lung cancer are non-small cell lung cancer (NSCLC) and small-cell lung cancer (SCLC). However, NSCLC is the most common form of lung cancer, accounting for 85% of all cases [2]. NSCLC is classified into different histological subtypes. The most common forms are lung adenocarcinoma (LUAD), lung squamous cell carcinoma (LUSC), and rarely large-cell carcinoma [3]. Although smoking is the major risk for lung cancer development [4], environmental factors, and genetic predisposition are also involved in cancerogenesis [5].

Detecting tumours early can lead to more effective treatment and increase overall survival (OS) rates. As a result, there are several efforts to diagnose lung cancer at an early stage in patients as part of cancer screening programmes [6]. The current standard for lung cancer screening is low-dose computed tomography (LDCT). However, it has limitations such as low specificity, radiation exposure and is a labour and equipment-intensive process [7]. A limited number of biomarkers are currently validated and used to predict the course of lung cancer or to select drug treatments. Exceptions include NSCLC patients with driver mutations (e.g., EGFR, ALK, ROS1, and MET) who are treated with targeted therapies [8].

The remaining patients with advanced-stage disease are given chemotherapies and/or immunotherapies (immune-checkpoint inhibitors, ICI) [9, 10]. However, there is a lack of simple, robust, and clinically relevant diagnostic, prognostic, and predictive biomarkers to tailor and personalise treatment strategies [11].

Recent research focus has shifted toward circulating biomarkers such as cell-free DNA (cfDNA) and various RNAs (long non-coding RNAs, lncRNA; microRNAs, miRNA). MiRNAs are small, non-coding RNAs that regulate post-transcriptionally gene expression in cells. Non-coding RNAs play crucial roles in cancer as tumour suppressors and oncogenes, and their dysregulation has been linked to NSCLC progression and metastasis [12–14]. MiRNAs are released by cells during apoptosis and necrosis, either actively or passively, and for cell-cell communication purposes. Traces of those released miRNAs eventually appear in the blood circulation [15].

For this study, we hypothesised that miRNAs overexpressed in various NSCLC subtypes (LUAD or LUSC) tissue would also be elevated in the blood circulation of patients. This pattern of elevated miRNA expression in the circulation could serve as a non-invasive diagnostic biomarker to identify NSCLC and distinguish between its subtypes. We aimed to investigate circulating miRNA as biomarkers in various clinical applications of NSCLC management and treatment. We investigated whether NSCLC-specific circulating miRNA, I) are elevated in the plasma of NSCLC patients compared to healthy individuals and could serve as a lung cancer non-invasive screening tool, II) could determine the prognosis of NSCLC patients, III) could be utilised as predictive markers for survival under ICI treatment, and IV) could assist in monitoring treatment response. We compared the circulating miRNA expression in the plasma of patients with LUAD and LUSC to healthy individuals, seeking diagnostic, prognostic, or predictive biomarkers. Our results were cross-validated using a publicly available independent dataset of nearly 4000 NSCLC patients, aiming to refine the use of miRNAs in NSCLC screening and improve patient survival. As a result, our findings advance the application of miRNAs as non-invasive biomarkers in NSCLC management and treatment personalisation.

## Methods

### Patients

The study included individuals >18 years of age with untreated, histologically proven diagnosis of NSCLC, irrespective of the disease stage or healthy individuals who served as controls. Participants provided informed consent to give blood for the study. Patients with previous treatment for lung cancer, such as surgery, radiotherapy, or systemic therapy, and patients with known secondary malignancies were excluded. Other types of non-cancerous diseases were not excluded. The Ethics Committee of the Canton of Bern, Switzerland approved the use of human specimens in this study (Project ID: 2018-01756). Blood samples were collected at Inselspital, University Hospital Bern, Switzerland.

### Specimen characteristics

Blood samples were collected in standard 7.5 mL EDTA tubes. Plasma was then extracted by centrifugation (20 °C, 7 min, 3100 x *g*), aliquoted into 0.5 mL cryotubes, and stored at −80 °C at the Liquid Biobank Bern, Inselspital, University Hospital, Bern.

### Assay methods

#### Identification of miRNA candidates

To screen miRNA candidates for the discovery cohort, we extracted the LUAD and LUSC tumour-matched data from TCGA Research Network: https://www.cancer.gov/tcga in RStudio v4.2.1 using the TCGAbiolinks/Bioconductor package [16]. Furthermore, miRNAs with low expression levels were excluded (only miRNAs that had normalised expression levels greater than 10 counts per million (CPM) in at least two different patient samples). The norm factor was calculated using edgeR’s calcNormFactor method “TMM” [17]. Differentially expressed miRNAs were determined according to the false discovery rate (FDR) < 0.05. Unsupervised clustering was performed on miRNAs with logFC >2 (4-fold induction or reduction).

#### MiRNA extraction and expression quantification

We used Qiagen’s miRNeasy Serum/Plasma Advanced Kit (Qiagen, Cat. No./ID: 217204) to extract miRNAs from a 0.5 mL plasma sample, following the manufacturer’s instructions, which included a spin down at 1000 rpm for 3 min and RNA extraction from the supernatant. The miRNA was extracted in a volume of 10 μL and used for reverse transcription. The Sp4/Sp5 spike-in mix was added at the beginning of the extraction as internal control. Sp4 was used as an extraction control and during the normalisation step (Qiagen, Cat. No./ID: 339390). The miRNA extraction sample (10 μL) was used for the reverse transcription transcribed with the miRCURY LNA RT kit (Qiagen, Cat. No./ID: 339340). Sp6 and Cel-miR-39-3p (Cel39) were added as an RT positive control during the RT process, according to the manufacturer’s instructions and recommendation (Qiagen, Cat. No./ID: 339390). Plasma-derived miRNA expression was measured using miRCURY LNA SYBR Green PCR kits (Qiagen, Cat. No./ID: 339345). MiRCURY LNA miRNA primers were used (Qiagen, Cat. No./ID: 339306). QPCR was conducted in 384-well plates with 10 μL end volume, 1:10 diluted cDNA using the Viia7 Real-Time PCR system (Applied Biosystems). ΔCt values were calculated using the Sp4 values (Ct(target)-Ct(Sp4)) (Qiagen, Cat. No./ID: 339390). Normalisation and statistical differences between LUSC and LUAD plasma and healthy donor samples were assessed using RStudio v4.2.1 (2022-06-23).

#### Haemolysis monitoring

Haemolysis contamination was defined as ΔCt (miR-23a-3p-miR-451a) > 7, as recommended by the miRCURY LNA miRNA Focus PCR Panels kit (Qiagen, Cat. No./ID: 339325).

#### Human CEA protein quantification

The concentration of Carcinoembryonic antigen (CEA) protein was measured using the RayBio^®^ Human CEA ELISA Kit (Cat. No./ID: ELH-CEA) with an initial 50 µL plasma according to the kit protocol.

### Study design

This study aimed to identify differentially expressed circulating miRNA expression in LUAD and LUSC subtypes of NSCLC and assess their potential as biomarkers for distinguishing between the subtypes of NSCLC and healthy donors. First, we selected the miRNAs that were pathologically upregulated in the cancer tissues of LUAD and LUSC patients compared to the normal adjacent tissues from The Cancer Genome Atlas (TCGA) data bank. Next, we evaluated the expression of those selected candidate miRNAs in the plasma of LUAD and LUSC cancer patients and healthy individuals serving as controls. We prospectively collected NSCLC and healthy plasma samples between 07-Dec-2018 and 29-Aug-2022. The end of follow-up was 04-Apr-2023, and the median follow-up time for survival in the entire NSCLC cohort was 43.2 months.

We assessed the time to progression (progression-free survival, PFS) and death (overall survival, OS). Collected clinicopathologic co-variables included: Disease stage according to the 8th edition of TNM classification, histological subtype, somatic genetic information, PD-L1 expression, smoking history, sex, age, applied treatment modalities, treatment response according to RECIST 1.1 criteria.

To investigate the performance of a miRNA-based diagnostic test for NSCLC detection, we calculated a sample according to these assumptions: Aimed test performance AUC = 0.75 AUC, null hypothesis AUC = 0.5, prevalence rate (the ratio of positive cases to the total sample size) = 0.66, type I error rate (alpha) = 0.05 and a power (1-beta) = 0.8. This resulted in a total sample size of 55 for each histological subtype (LUAD and LUSC), comprising 36 cancer patients and 19 healthy controls. We also factored in a potential dropout rate of up to 25% due to issues related to sample quality or other technical or medical complications. As a result, we aimed to include a minimum of 138 patients in the study [18].

All procedures performed in this study involving human participants were in accordance with the ethical standards of the institutional and/or cantonal ethics committee, with the Helsinki Declaration and with the Swiss Federal Human Research Act (HRA).

### Statistical analysis methods

To optimise miRNA combinations for enhanced diagnostic accuracy, the best-performing miRNA combinations for NSCLC detection were calculated using the CombiROC algorithm [19]. The CombiROC algorithm selects the miRNAs with the highest area under the curve (AUC) in a receiver operating characteristic (ROC) curve and displays their test sensitivity (SE) and specificity (SP) values.

The best-performing miRNA panels were validated with independent, publicly available datasets of both NSCLC and pneumonia patients [20, 21].

Differential expression analysis and Cox regression were performed using RStudio v4.2.1 (2022-06-23) and GraphPad Prism v10 (GraphPad Software, USA). Data was analysed using a one-way-ANOVA and two-tailed Student’s t-test. Survival time differences were plotted using Kaplan–Meier curves and analysed using the log-rank test. The OS cut-offs based on the expression of DE miRNAs were assessed using the X-tile programme [22]. Details on the quantification, normalisation, and statistical tests used in every experiment can be found in the corresponding figure legend. All *p*-values were considered significant when *p* < 0.05. Data are displayed as mean ± SD. We clustered the data for heatmaps according to Euclidean’s method based on the average linkage and generated heatmaps according to the standard normal distribution.

## Results

**Fig. 1:**
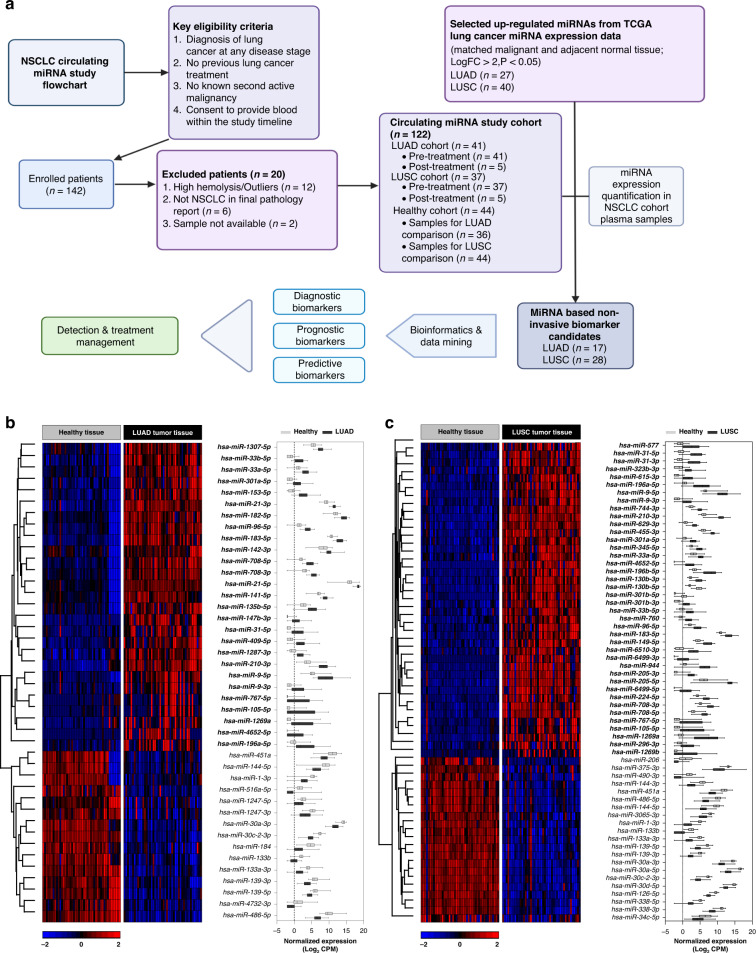
Overview of study design and miRNA selection in LUAD and LUSC NSCLC subtypes from TCGA data. **a** Flow chart for the NSCLC circulating miRNA study. **b** Heatmap and boxplot showing the differentially expressed (DE) miRNAs in malignant lung cancer and matched normal tissue. Rows represent miRNA IDs; columns represent TCGA sample IDs. A log_2_FC > 2 (4-fold induction or reduction) with a *p*-value of <0.05 was used as a threshold and considered significant. Data is presented as means ± SD. In LUAD, 42 miRNAs are differentially expressed (27 upregulated, 15 downregulated in cancer tissue). **c** In LUSC, 61 miRNAs are differentially expressed (40 upregulated, 21 downregulated in cancer tissue).

In this translational, single-centre, cohort study, we enrolled 142 individuals to quantify and compare cancer-derived miRNAs in the plasma of NSCLC patients and healthy donors (Fig. 1a). Patients with newly diagnosed NSCLC before beginning treatment who consented to provide blood were included. Out of the 142 enrolled study participants, 20 were excluded from the analysis due to sample issues or diagnostic reasons, including high haemolysis or outliers with mean miRNA expression of greater than three standard deviations (*n* = 12), non-NSCLC in final pathology report (*n* = 6) and loss of sample (*n* = 2). Finally, 122 participants, including 41 LUAD, 37 LUSC, and 44 healthy individuals, were included in the study.

### Patient’s characteristics

The baseline characteristics of enrolled patients are summarised in Supplementary Table 1. Our study population includes patients with early and late-stage NSCLC. However, late-stage patients are predominant. The median age of the patients did not differ significantly; however, the LUSC cohort had a higher number of men and a higher smoking rate than the LUAD cohort, reflecting the strong association of male smokers with the incidence of LUSC.

### Comparative analysis of miRNA profiles in NSCLC and adjacent normal tissues of LUAD and LUSC

We conducted a miRNA differential expression (DE) analysis based on 46 LUAD or 45 LUSC and their matched adjacent normal lung tissue samples using data from The Cancer Genome Atlas (TCGA) database [16]. Analysing the LUAD tissue dataset revealed 27 upregulated and 15 downregulated miRNAs (log_2_FC < −2, *p* < 0.05) relative to normal lung tissue (Fig. 1b). The LUSC cohort contained 40 upregulated and 21 downregulated miRNAs (Fig. 1c). We discovered only a subset of miRNAs (16 upregulated and 10 downregulated) that were deregulated in both NSCLC subtypes (Supplementary Fig. 1A, B). These results indicate that the different NSCLC subtypes have distinct miRNA expression signatures, implying that different miRNA panels need to be investigated for NSCLC biomarker studies. We then selected the DE miRNAs from LUAD and LUSC to investigate their presence in the bloodstream.

### Differences in plasma miRNA profiles between NSCLC patients and healthy individuals: potential for non-invasive diagnostic miRNA-based biomarkers

**Fig. 2:**
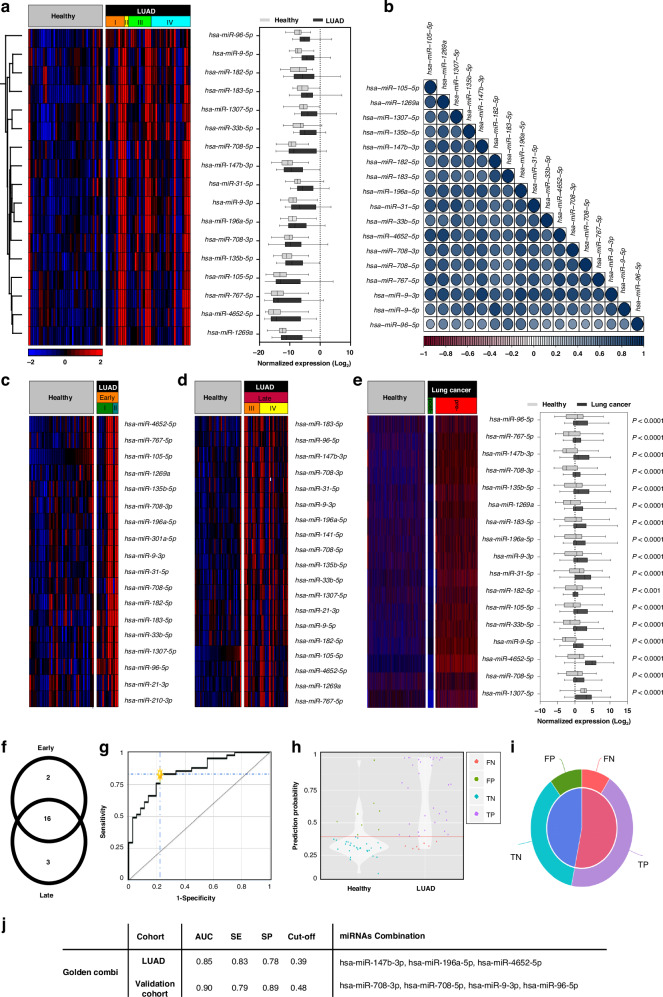
Comparative analysis of plasma-derived miRNAs in LUAD patients and healthy individuals. **a** Heatmap and boxplot indicate DE miRNA expression (*n* = 17). **b** Correlation plot of DE miRNA expression levels. Similar variables are placed adjacently using correlation-based ordering. Darker colours and larger circles indicate stronger correlations. Blue indicates a positive correlation, while red represents a negative correlation. **c** Heatmap and boxplot of DE miRNA expression (*n* = 17) in the validation cohort. Data is displayed as means ± SD; statistical evaluation using Student’s *t*-test and significant are miRNAs with *p* < 0.05. **d** Heatmap of DE miRNA expression in early disease stage (*n* = 18). **e** Heatmap of DE miRNA expression in the late disease stage (*n* = 19). **f** Venn diagram depicting overlapped DE miRNA (*n* = 16) between early-stage vs. healthy (*n* = 18) and late-stage vs. healthy (*n* = 19), as well as stage-specific miRNAs in the early-stage (*n* = 2) and late-stage (*n* = 3). **g** Receiver operating characteristic (ROC) analysis reveals the best combination panel of DE miRNAs with the highest sensitivity (SE) and specificity (SP), as well as the best area under the curve (AUC) for the LUAD cohort. **h** Violin plot shows the probability density for the two compared sample groups (LUAD vs. healthy). **i** Pie chart shows the percentages of false predictions (false positives, FPs; false negatives, FNs) and true predictions (true positives, TPs; true negatives, TNs). **j** Table displays the best miRNA combination panel according to the highest AUC, SE%, SP%, and optimal cut-off in both LUAD and Validation validation cohorts, as determined by the CombiRoc analysis.

**Fig. 3:**
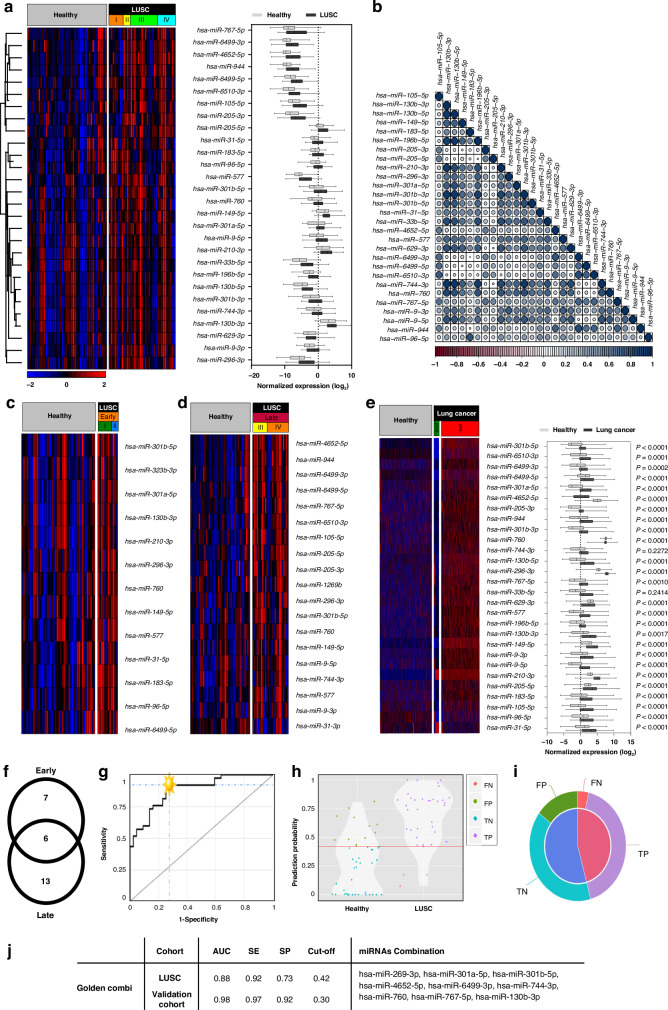
Comparative analysis of plasma-derived miRNAs in LUSC patients and healthy individuals. **a** Heatmap and boxplot indicate DE miRNA expression (*n* = 28). **b** Correlation plot of DE miRNA expression levels. Similar variables are placed adjacently using correlation-based variable ordering. Darker colours and larger circles indicate stronger correlations. Blue indicates a positive correlation, while red represents a negative correlation. **c** Heatmap and boxplot show DE miRNA expression (*n* = 28) in the validation cohort. Data is displayed as means ± SD; statistical evaluation using Student’s *t*-test and significant are miRNAs with *p* < 0.05. **d** Heatmap of DE miRNA expression in early disease stage (*n* = 13). **e** Heatmap of DE miRNA expression in late disease stage (*n* = 19). **f** Venn diagram depicting overlapped DE miRNA (*n* = 6) between early-stage vs. healthy (*n* = 13) and late-stage vs. healthy (*n* = 19), as well as stage-specific miRNAs in the early-stage (*n* = 7) and late-stage (*n* = 13). **g** ROC analysis reveals the best combination panel of DE miRNAs with the highest sensitivity (SE) and specificity (SP), as well as the best area under the curve (AUC) for the LUSC cohort. **h** Violin plot shows the probability density of the two compared sample groups (LUSC vs. healthy). **i** Pie chart shows the percentages of false predictions (false positives, FPs; false negatives, FNs) and true predictions (true positives, TPs; true negatives, TNs). **j** Table displays the best miRNA combination panel according to the highest AUC, SE, SP, and optimal cut-off in both LUSC and validation cohorts as determined by the CombiRoc analysis.

We investigated the expression of the miRNA candidates in the plasma of NSCLC patients to identify miRNAs that could be used as diagnostic biomarkers to distinguish NSCLC patients from healthy individuals. We focused on overexpressed miRNA because we hypothesised that those miRNAs are highly released into the circulation and could be reliably detected, whereas downregulated miRNAs in cancer cells do not affect a patient’s total pool of circulating miRNA. We discovered that 17 of 27 miRNA candidates in LUAD and 28 of 40 in LUSC were significantly upregulated in the plasma of our NSCLC cohort. The expression levels of DE circulating miRNAs are shown in Figs. 2a and  3a. The expression of all miRNA candidates for both studied cohorts is available in Supplementary Fig. 2A, Supplementary Fig. 3A, and Supplementary dataset.

The correlation between DE miRNAs in NSCLC subtypes is shown in Figs. 2b and 3b. In LUAD, DE miRNA expression was strongly correlated, whereas, in LUSC, individual miRNA expression patterns varied more widely. Furthermore, we validated our DE miRNA expression in an independent, publicly available dataset with blood samples from more than 4000 lung cancer patients [21], which confirmed significantly higher expression of our DE miRNA in the blood of NSCLC patients compared to healthy individuals for both LUAD and LUSC cohorts (Figs. 2e and  3e). Further, the validation cohort included an additional blood sample analysis after surgical removal of the lung cancer and in nearly all samples, a significant drop in the miRNA expression was observed. This suggests that the circulating miRNAs originate from the tumour.

We investigated whether some miRNAs are expressed in a stage-specific manner and analysed early (stage I and II) and late tumour stages (stage III and IV), respectively (Figs. 2c, d and  3c, d). The overlap of stage-specific miRNAs is shown in Figs. 2f and  3f. Overall, miRNA expression levels in LUAD were highly independent of tumour stage. Only hsa-miR-210-3p and hsa-miR-301a-5p were identified as early-stage biomarkers, and hsa-miR-9-5p, hsa-miR-141-5p, and hsa-miR-147b-3p as late-stage biomarkers. In LUSC, we observed a higher variety between early and late-stage-specific miRNA, as only six miRNA species appeared in both stages. Of note, hsa-miR-210-3p and hsa-miR-301a-5p appeared as early-stage-specific miRNAs in both LUAD and LUSC cohorts, and hsa-miR-9-5p was found to be a late-stage biomarker in both cohorts.

As a standard clinical procedure, the tumour tissue of our LUAD stage IV patients was tested for driver mutations. We identified mutations in *EGFR* (*n* = 4), *KRAS* (*n* = 7), *ALK* (*n* = 3), and copy number gain in *MET* (*n* = 1). Two miRNAs (has-miR33b-5p and has-mir-9-3p) were significantly upregulated in *ALK*-mutated patients compared to the other patients. In contrast, in the plasma of a few *EGFR*-mutated patients, the miRNA candidates were not upregulated. Five miRNA candidates were even significantly downregulated in comparison to the other cancer samples (Supplementary Fig. 2B).

Next, we performed a CombiROC analysis to determine the best-circulating miRNA panel for detecting NSCLC in the plasma. The CombiROC algorithm calculates a set of miRNAs that produce the largest area under the curve (AUC) in a receiver operating characteristic (ROC) curve and displays the corresponding test sensitivity (SE) and specificity (SP) values [19]. We performed this analysis separately for LUAD and LUSC cohorts (Fig. 2g–i and  3g–i). We found the best combination consisted of seven miRNA species with AUC = 0.85, SE = 83%, and SP = 78% in the LUAD cohort (Fig. 2j), and nine miRNAs with AUC = 0.88, SE = 92%, and SP = 73% in the LUSC cohort (Fig. 3j). The diagnostic performance of these miRNA combinations was then verified in the publicly available validation cohort [21]. Both the LUAD and the LUSC miRNA combination showed improved performance in the validation cohort, achieving an area under the curve (AUC) values of 0.90 and 0.98, respectively (Figs. 2j and  3j). This indicates that the expression values of the miRNAs that we selected have diagnostic potential. The miRNA combinations with the highest sensitivity or specificity, independent of AUC, are listed in Supplementary Fig. 2C–E and Supplementary Fig. 3B–D.

Furthermore, we investigated the performance of the DE miRNAs overlapped by LUAD and LUSC (*n* = 9). The CombiROC analysis of the entire NSCLC cohort identified a panel of seven miRNA combinations as the best performing (AUC = 0.83, *S* = 73%, SP = 81%). The combination does not reach the same sensitivity and specificity as subtype-specific ones; however, we also observe a better test performance in the validation cohort (AUC = 0.91, SE = 83%, SP = 86%) (Supplementary Fig. 4A–F). The miRNA combinations with the highest sensitivity or specificity, independent of AUC are listed in Supplementary Fig. 4G–I.

We also assessed the expression of our diagnostic miRNA panel for LUAD and LUSC in a publicly available dataset of plasma samples from patients with community-acquired pneumonia (CAP) as a representative non-cancerous lung disease [20]. Six out of seven miRNAs from the LUAD panel and five out of nine miRNAs from the LUSC panel were assessed. The majority of the miRNAs identified in NSCLC exhibited significantly different expression levels compared to CAP (Supplementary Fig. 5A–D). However, a few miRNAs were also elevated in CAP, underscoring the critical role of utilising miRNA panels to maximise the specificity for NSCLC diagnosis.

To better assess the value of our miRNA panels, we also evaluated a representative subset of our study cohort using an established tumour marker such as Carcinoembryonic Antigen (CEA) [23]. However, when we quantified plasma CEA levels in our LUAD and LUSC cohort, we did not observe a significant increase in CEA levels among LUAD and LUSC patients compared to healthy individuals. This finding underscores the sensitivity and robustness of our identified miRNA-based panel for diagnosing NSCLC (Supplementary Fig. 5E).

### Circulating miRNAs serve as prognostic biomarkers for NSCLC

**Fig. 4:**
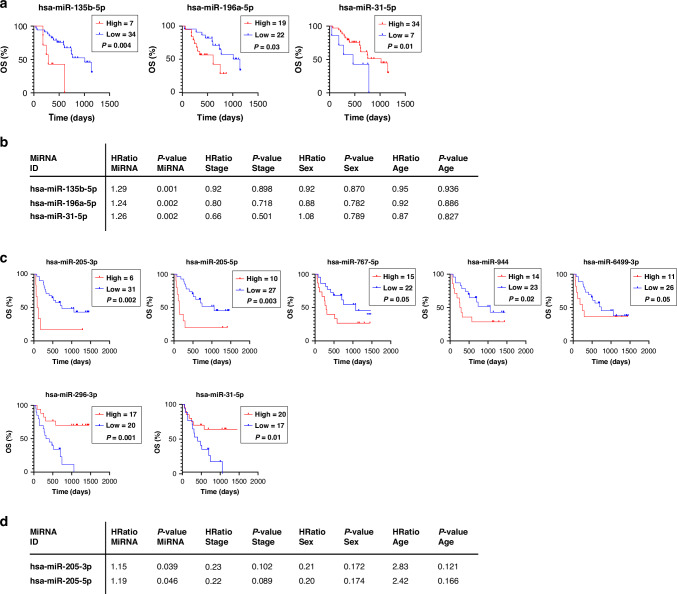
A panel of differentially expressed miRNAs serves as Non-Invasive prognostic biomarkers in NSCLC. **a** Kaplan–Meier plots of overall survival (OS) for the LUAD cohort showing significant prognostic miRNAs based on DE miRNA expression (*n* = 3, Log-rank test *p* < 0.05). The cut-off for high or low miRNA expression was assessed by the X-tile programme. **b** Multivariate Cox regressional hazard analysis for prognostic miRNAs (*n* = 3) in LUAD cohort with clinical variables such as stage, sex, and age (NS: non-significant, HRatio: hazard ratio). **c** Kaplan–Meier plots of OS for the LUSC cohort representing significant prognostic miRNAs according to DE miRNA expression (*n* = 7, Log-rank test *p* < 0.05). The cut-off for high or low miRNA expression was assessed by the X-tile programme. **d** Multivariate Cox regressional hazard analysis for prognostic miRNAs (*n* = 2) in the LUSC cohort with clinical variables such as stage, sex, and age (NS non-significant, HRatio hazard ratio).

We performed a Kaplan–Meier survival analysis to determine which circulating miRNAs predict the OS and could work as a prognostic biomarker. We defined optimal cut-off points for separating patients into high and low miRNA-expressing groups using the X-tile programme [22, 24–26]. In the LUAD cohort, we identified three statistically significant miRNAs (hsa-miR-135b-5p, hsa-miR-196a-5p, hsa-miR-31-5p) as prognostic biomarkers. High expression of hsa-miR-135b-5p and hsa-miR-196a-5p was a poor prognostic marker, and high expression of hsa-miR-31-5p was associated with a better prognosis (Fig. 4a). Next, we performed multivariate Cox regressional hazard analysis to test what miRNAs are prognostic independent of clinical variables such as sex, age, and tumour stage (Fig. 4b). Three of the significant miRNAs from the X-tile analysis appeared as independent prognostic biomarkers in LUAD.

In LUSC, we identified seven miRNAs (hsa-miR-205-3p, hsa-miR-205-5p, hsa-miR-767-5p, hsa-miR-944, hsa-miR-6499-3p, hsa-miR-296-3p, hsa-miR-31-5p) as prognostic biomarkers (Fig. 4c). Remarkably, both strands of miR-205 appear to have a prognostic value. High expression of hsa-miR-31-5p appears to be prognostically favourable in both histological subtypes of NSCLC. In the LUSC cohort, the multivariate Cox regression hazard analysis revealed only two independent prognostic biomarkers (hsa-miR-205-3p, hsa-miR-205-5p) (Fig. 4d). This is primarily due to the higher stage dependence of miRNA expression in LUSC, as shown in Fig. 3f.

To summarise, our results indicate that hsa-miR-135b-5p, hsa-miR-196a-5p, and hsa-miR-31-5p for LUAD, as well as both strands of hsa-miR-205, are promising prognostic biomarkers that should be further validated in other independent cohorts prospectively. The insignificant miRNAs for both LUAD and LUSC are shown in Supplementary Fig. 6.

### Circulating miRNA as non-invasive predictive biomarkers for anti-PD-1 immune-checkpoint inhibitor therapy in NSCLC

We investigated whether circulating miRNA could predict survival under immunotherapy with an anti-PD1 immune-checkpoint inhibitor (ICI). We performed a Kaplan–Meier survival analysis on 12 LUAD patients who received ICI alone (ICI mono; *n* = 5) or ICI plus chemotherapy (ICI + Chemo; *n* = 7) as first-line treatment for advanced-stage disease. Due to the low number of LUSC patients that received ICI treatment (*n* = 4), this cohort could not be analysed. We chose PFS after the start of ICI treatment as the endpoint to eliminate possible bias by second-line therapies.

**Fig. 5:**
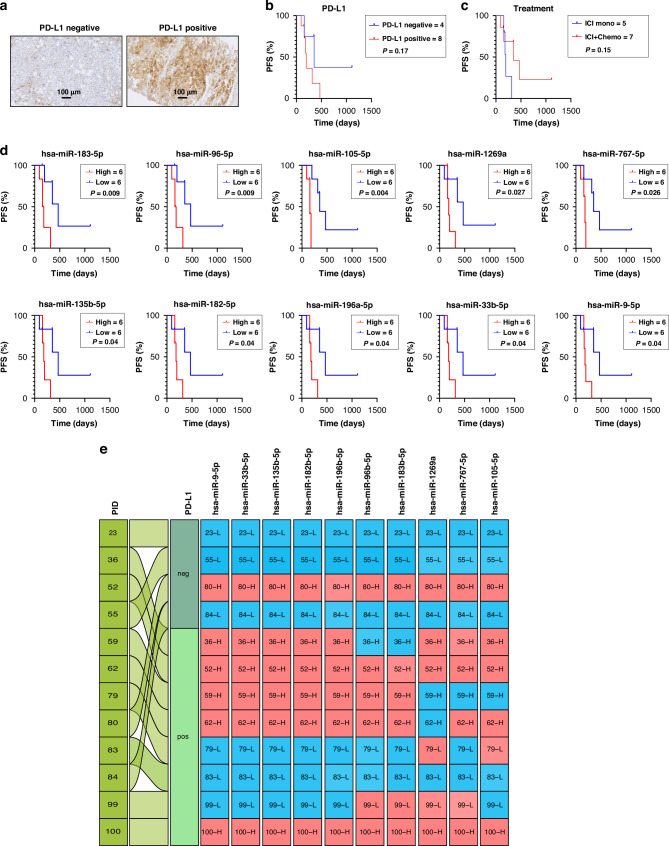
Elucidating miRNA profiles as predictive biomarkers in NSCLC upon anti-PD-1 immunotherapy. **a** PD-L1 tissue staining (positive >1%, negative <1%). **b** Tissue PD-L1 expression-based Kaplan–Meier-estimated progression-free survival (PFS) in NSCLC patients (*n* = 12). **c** Kaplan–Meier-estimated PFS in NSCLC patients treated with ICI mono (*n* = 5), and ICI + Chemotherapy (*n* = 7). **d** Kaplan–Meier-estimated PFS based on DE miRNAs (*n* = 10) in NSCLC patients treated with ICI. **e** The alluvial plot illustrates the patient cohorts undergoing ICI therapy. These groups are split into two categories: PD-L1 negative or positive. Within this framework, ‘H’ represents a high expression of miRNA, quantified as exceeding the median expression levels showed as a red colour, while ‘L’ represents a low expression of miRNA, situated below the median threshold, showed as a blue colour.

Currently, PD-L1 expression in tumour tissue is used as a predictive marker for ICI treatment. However, immunohistochemistry-measured PD-L1 is regarded as a controversial and semi-reliable predictive biomarker for ICI treatment [27]. Our studied cohort contained four PD-L1 negative ( < 1%) and eight PD-L1 positive ( ≥ 1%) tissue samples (Fig. 5a). Indeed, PD-L1 expression in tissue was not predictive of survival (Fig. 5b). Furthermore, there was no difference in outcome between patients who received ICI monotherapy or ICI plus chemotherapy combination treatment (Fig. 5c).

We assessed the predictive value of each individual DE miRNA by categorising LUAD patients into two groups (low and high expression) using the median miRNA expression as a cut-off. We discovered that 10 out of 28 DE miRNAs were predictive for PFS under ICI treatment (Fig. 5d). Specifically, hsa-miR-105-5p and hsa-miR-767-5p, showed the highest significance for survival prediction upon ICI treatment. A pattern of long versus short therapy responders emerged, indicating that patients with a high amount of one predictive circulating miRNA also have a high amount of expression in the other miRNAs (Fig. 5e). Those “miRNA-shedders” have a lower survival rate under ICI treatment than patients with lower expression levels.

### Monitoring treatment responses in lung cancer patients can be achieved through the analysis of circulating miRNA expression

**Fig. 6:**
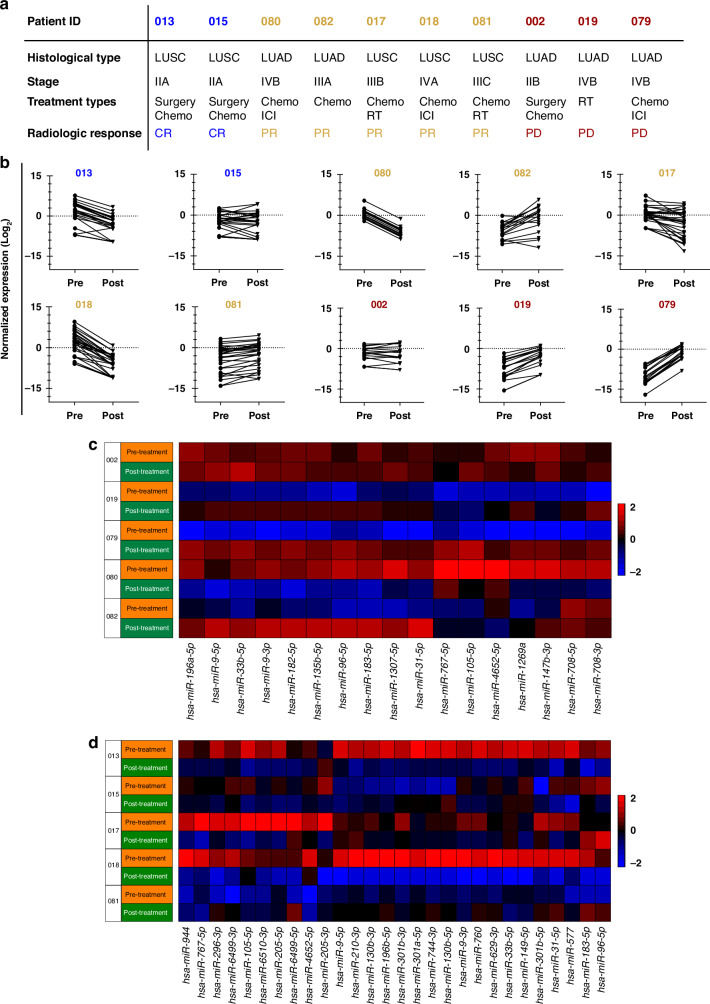
Assessing treatment response through comparative miRNA profiling before and after NSCLC therapy. **a** Clinical profiles of patients treated with various therapies (*n* = 10) (CR: Complete Response, blue; PR partial response, yellow; and PD progressive disease, red) in the second blood withdrawal. **b** Changes in DE miRNA expression alteration between pre-treatment and post-treatment samples. **c** Heatmap of 17 differentially expressed miRNAs in five LUAD patients. **d** Heatmap of 28 differentially expressed miRNAs in five LUSC patients.

We investigated whether repetitive measurement of circulating miRNA could be used to monitor lung cancer therapy response. Ten patients had a second blood draw approximately 90 days after starting treatment (range 63–126 days), in which the DE miRNAs were measured again. To assess tumour response according to RECIST 1.1 criteria, we used the radiological examination result that was closest in time to the second blood draw. The average time between blood draw and radiological examination was 13 days (Fig. 6a). We observed a clear trend in most samples where a decrease or increase in circulating miRNAs correlated with radiologic responses (Fig. 6b). The individual change of miRNAs in each sample is illustrated for both LUAD and LUSC patients in Fig. 6c, d. However, in some samples, we discovered that the trend was not evident in all analysed miRNAs (patients #002, 015, 017, 081) or in one sample, an increase in DE miRNAs was observed despite radiologic tumour response to chemotherapy (#082). However, patient 082 with NSCLC Stage IIIA received neoadjuvant treatment, and blood samples were taken before and after neoadjuvant chemotherapy. Six months after surgery, the patient experienced a tumour relapse, indicating that chemotherapy’s effectiveness was limited. These results suggest that the expression profile of circulating miRNA reflects changes in patients’ tumour burden, and those measurements can be used to monitor treatment responses.

## Discussion

In this study, we demonstrate that circulating miRNA can be a source of novel biomarkers for the diagnosis and treatment management of NSCLC. In our study approach, we combined different data sources to identify the most robust and relevant biomarkers: starting with publicly available TCGA data on miRNA expression in tumour tissue, we focused on a limited number of miRNA candidates to minimise non-tumour-related influences on the composition of the circulating miRNA pool. We then investigated these miRNA candidates in a prospective cohort of NSCLC patients and healthy individuals at our Cancer Centre, and our findings were validated in an independent, previously published big study cohort of more than 4000 lung cancer patients.

Our targeted screening approach differs from other studies that have investigated circulating miRNA as diagnostic markers for lung cancer. In these studies, the entire miRNA profile of a sample is first analysed using a microarray, and then the expression is compared between diseased and healthy samples [21, 28]. The simplicity of our targeted approach, as well as the lower number of candidate miRNAs for testing, may provide advantages in terms of cost-effectiveness and rapid qPCR analysis. Such an approach is easily adaptable to clinical applications. Furthermore, we have a high certainty that the investigated miRNA originates from the tumour, which may lead to improved diagnostic specificity. This approach also opens the possibility of developing a therapy-guiding diagnostic tool for future miRNA-based treatment approaches.

There are currently several approaches for detecting lung cancer at an early stage by screening high-risk patients, such as smokers, to improve survival (e.g., low-dose CT, ctDNA) [7, 29]. Some miRNA species from our diagnostic panel have already been investigated as biomarkers of NSCLC (Supplementary Table 3). We demonstrate that circulating miRNA can be used to diagnose both LUAD and LUSC, with some miRNAs appearing in both histological subtypes while others are specific. If these results are to be used in a clinical setting, both scenarios, a panel with common miRNA as well as subtype-specific panels, could be valuable. The fact that we confirmed our results in an independent cohort with a different starting material (serum instead of plasma) and a different screening assay (microarray) [21], gives us confidence that our diagnostic panels are reliable and reproducible. To implement such a diagnostic test for NSCLC screening in clinical practice, proximity to at-risk patients is crucial. Strategies include low-threshold testing endorsed by family doctors or pneumologists. Integrating the blood test into national cancer screening programmes, perhaps alongside existing CT scans, could further enhance acceptance among patients and primary care providers.

The strength of our study is the linkage of circulating miRNA expressions with clinicopathological data. Individual miRNAs such as hsa-miR-135b-5p, hsa-miR-196a-5p, hsa-miR-31-5p, and hsa-miR-205 appear to be prognostic biomarkers independent of tumour stage, sex, and age. Our study establishes a link between findings from tissue studies and circulating miRNA. For example, recent research demonstrates a correlation between hsa-miR-135b-5p in lung cancer tissue and poor patient survival [30]. Furthermore, hsa-miR-205 has been extensively investigated in various studies and is a well-known marker for squamous cell carcinomas [31, 32]. However, there is contradictory evidence regarding the prognostic significance of hsa-miR-205 in tumour tissue [33]. In our study, increased expression of circulating hsa-miR-205 in LUSC was associated with poor survival, as previously shown in another study [34]. Prognostic markers can assist in identifying patients who may require more intensive treatment. For example, adjuvant chemo- and immunotherapy after surgery improves survival, particularly in stage II/III NSCLC [34, 35]. Nevertheless, the absolute benefit of adjuvant therapy remains limited. A more targeted selection of patients who can truly benefit from adjuvant therapy remains desirable. Our prognostic markers may help identify patients who are at high or low risk of relapse. The applicability of circulating miRNA in the selection of adjuvant therapies must be tested in an independent study.

We investigated whether individual circulating miRNA levels can predict PFS of patients treated with anti-PD-1 ICI. Indeed, 10 upregulated miRNAs in the plasma are associated with a shorter PFS under ICI treatment. We identified the expression of two miRNA clusters associated with PFS (on chromosome 7: hsa-miR-183, hsa-miR-96, hsa-miR-182; and on chromosome X: hsa-miR-105, hsa-miR-767). Some of the important miRNAs have already been associated with immunosuppressive effects: Cancer-exosomes derived hsa-miR-9 has been shown to induce early myeloid-derived suppressor cells (eMDSCs), which trigger apoptosis and inhibit proliferation of T-cells [36]. In gastric cancer, exosomal miR-135b-5p impairs the function of intratumoral γδ T-cells [37]. Upregulation of hsa-miR-183 through TGF-beta signalling inhibits Natural killer cells [38]. Hence, the impact of these miRNAs on the tumour microenvironment and immune cells was beyond the scope of the present study.

In contrast to other tumour entities, such as prostate-specific antigen (PSA) in prostate cancer, there are no widely established biomarkers in the clinic for monitoring treatment response in NSCLC. Radiographic exams (computed tomography) are commonly used to determine treatment response under therapy or remission status following curative treatment. Those radiologic examinations must be repeated at longer intervals to provide information on response. They are resource-intensive and expose patients to radiation. Liquid biomarkers, which are non-invasive, could provide a rapid assessment of treatment response or remission status and support clinical treatment decisions. In our analysis, we found that serial measurement of the candidate miRNAs correlates with tumour response according to radiological criteria in the vast majority of cases. This finding suggests that measuring circulating miRNA with cost-effective laboratory methods for universal adoption, such as qPCR, allows monitoring treatment responses in NSCLC in a non-invasive manner.

In summary, our study offers several advantages over existing analyses: (I) Targeted miRNA candidate selection: Our innovative approach, which involves selecting specific miRNA candidates from tissue analysis, can be applied to other tumour types. (II) Simple analysis method: Our straightforward analysis method by qPCR requires minimal effort to implement in clinical practice. (III) Validation: We validated our diagnostic miRNA panels in completely independent data sets. (IV) Integration of clinical parameters: we provide a comprehensive analysis of circulating miRNAs, spanning different levels such as diagnosis, prognosis, therapy response, and disease progression. Our study has a few limitations: Normalising circulating miRNA against endogenous genes is known to be challenging. To moderate this issue, we used uniform plasma volumes and normalised against RNA spike-ins. In this study, we did not investigate whether the miRNA circulates freely or in extracellular vesicles. Our findings in diagnosis, particularly in survival prediction during ICI treatment, are based on a small cohort of patients. These findings should be validated thoroughly in a larger study cohort.

In summary, the findings support that circulating miRNAs have enormous potential as biomarkers in the management of NSCLC patients, from diagnosis to treatment and monitoring (Supplementary Tables 2 and Table 3).

## Conclusion

Our study highlights the substantial clinical potential of circulating miRNA profiling as a robust and non-invasive tool for managing NSCLC. We provide validated circulating miRNA profiles specifically tailored for NSCLC diagnosis, demonstrating high sensitivity and specificity. Furthermore, we present evidence supporting individual miRNAs as both independent prognostic biomarkers and predictors of response to immune-checkpoint inhibitors.

## Supplementary information

Supplementary Figures and Tables

Supplementary Dataset (Differentially expressed miRNAs)

## Data Availability

All human transcriptomic data compiled for this study was taken from the Gene Expression Omnibus (GEO) (https://www.ncbi.nlm.nih.gov/geo/) database with accession number GSE137140.
