# Supplementary material for: Circulating miRNA panels as a novel non-invasive diagnostic, prognostic, and potential predictive biomarkers in non-small cell lung cancer (NSCLC)
Source: Br J Cancer. 2024 Aug 30;131(8):1350–62. doi: 10.1038/s41416-024-02831-3 (PMC11473829; doi:10.1038/s41416-024-02831-3)
Supplement: Supplementary file 1 — Supplementary Figures and Tables [file 41416_2024_2831_MOESM1_ESM.pdf]

Supplementary Figures and Tables

Circulating miRNA Panels as a Novel Non-Invasive Diagnostic, Prognostic, and Potential Predictive Biomarkers in Non-Small Cell Lung Cancer (NSCLC)

Authors:

Maryam Abdipourbozorgbaghi[1,2,3], Adrienne Vancura[1,2,3], Ramin Radpour[1,2#*], Simon Haefliger[1,2#*]

Affiliations:

[1]Department of Medical Oncology, Inselspital, Bern University Hospital, University of Bern, Bern, Switzerland.

[2]Department of BioMedical Research (DBMR), University of Bern, Bern, Switzerland.

[3]Graduate School of Cellular and Biomedical Sciences, University of Bern, Bern, Switzerland.

# Joint senior authorship

* Corresponding authors

Supplementary Figures

- Supplementary Figure 1. Overlapped miRNA profiles in diverse NSCLC subtypes.
- Supplementary Figure 2. Comparison of plasma-derived miRNAs in LUAD patients and healthy individuals.
- Supplementary Figure 3. Comparison of plasma-derived miRNAs in LUSC patients and healthy individuals.
- Supplementary Figure 4. Panel of differentially expressed overlapped miRNAs serve as non-invasive diagnostic biomarkers in NSCLC.
- Supplementary Figure 5. Evaluation of differentially expressed miRNAs in NSCLC vs. CAP and comparison of CEA protein concentration.
-
- Supplementary Figure 6. Prognostic value of differentially expressed miRNAs as non-invasive biomarkers for NSCLC Subtypes.

Supplementary Tables

- Supplementary Table 1. Patient characteristics in NSCLC and healthy individuals.
- Supplementary Table 2. Summary of the identified miRNA-based diagnostic, prognostic, and predictive biomarkers for NSCLC subtypes.
- Supplementary Table 3. Summary of the identified miRNA-based diagnostic biomarkers for NSCLC subtypes in different studies.

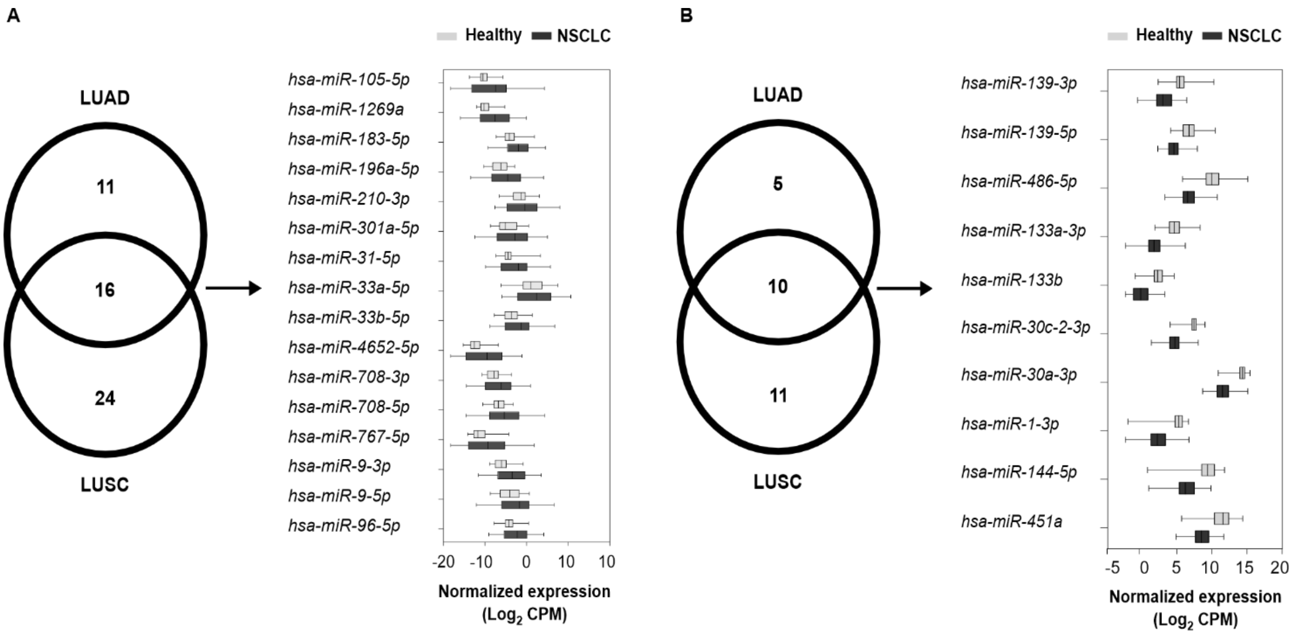

Supplementary Figure 1. Overlapped miRNA profiles in diverse NSCLC subtypes.

A) Venn diagram depicting overlap upregulated miRNAs (n=16) between LUAD (n=27) and LUSC (n=40) subtypes,boxplot showing the expression of those overlapped miRNAs (n=16) in NSCLC and healthy cohort. Data are displayed as means ±SD. B) Venn diagram depicting overlap downregulated miRNAs (n=10) in LUAD (n=15) and LUSC (n=21) subtypes,boxplot showing the expression of those overlapped downregulated miRNAs (n=10) in NSCLC and healthy cohort. Data are displayed as means ±SD.

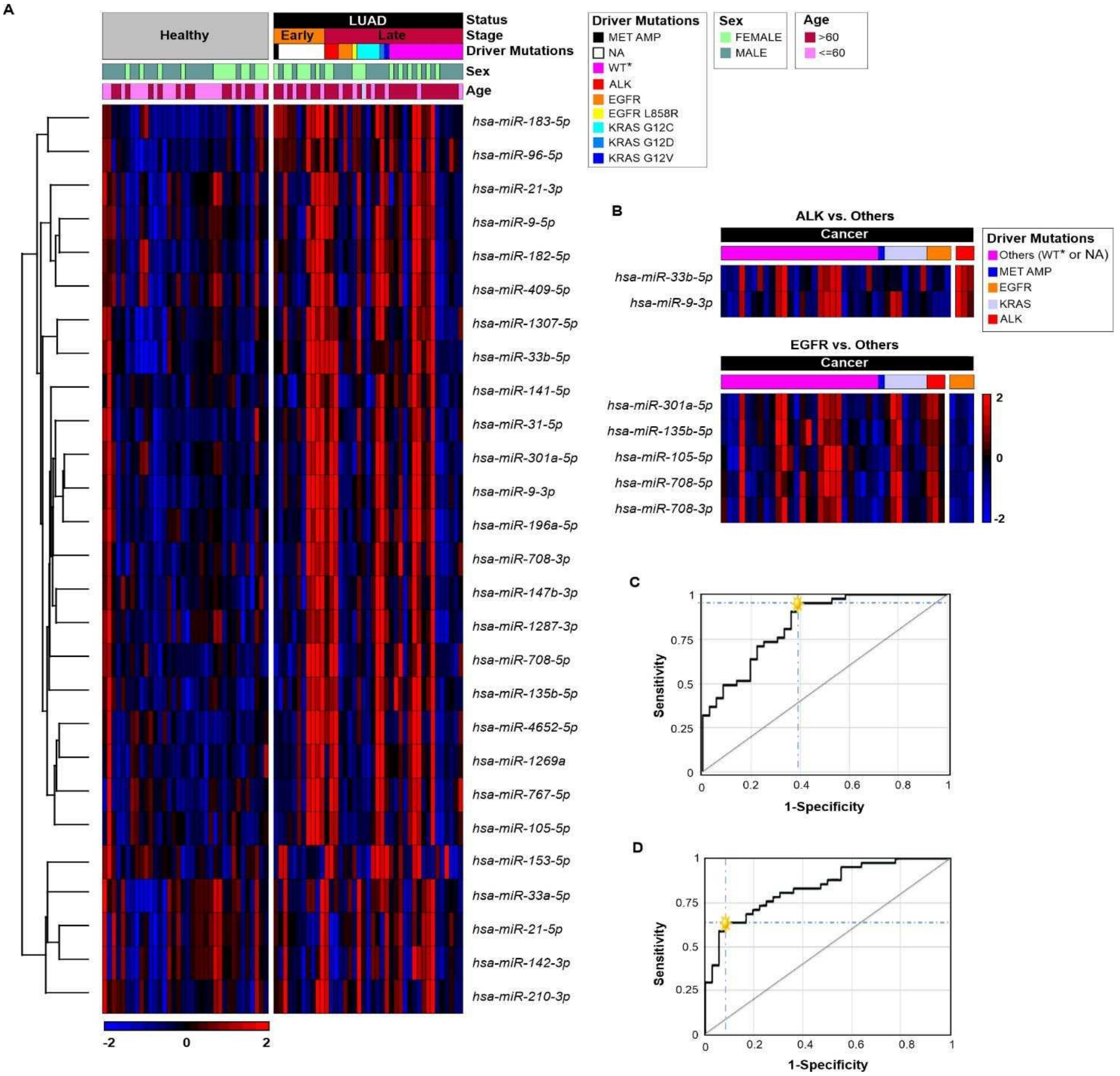

E

	Type	AUC	SE	SP	Cut-off	miRNAs Combination
LUAD Combi	Highest SE	0.84	0.95	0.61	0.36	hsa-miR-1269a, hsa-miR-183-5p, hsa-miR-196a-5p, hsa-miR-708-3p, hsa-miR-708-5p, hsa-miR-9-3p
	Highest SP	0.84	0.63	0.92	0.49	hsa-miR-1269a, hsa-miR-96-5p, hsa-miR-196a-5p, hsa-miR-708-3p, hsa-miR-708-5p, hsa-miR-9-3p

Supplementary Figure 2. Comparison of plasma-derived miRNAs in LUAD patients and healthy individuals.

A) Heatmap shows the expression of all analyzed LUAD miRNAs (n=27) with a $\log_2$ fold change of ≥2 and a significance level of $P<0.05$ arranged according to clinical characteristics (stage, mutations, sex, and age). B) Heatmap of DE miRNAs stratified by driving mutations: This includes *ALK*$^{+}$ (n=3) compared to other mutation subgroups comprising *EGFR*$^{+}$ (n=3), *EGFR* ^{L858R} (n=1), *KRAS*^{G12C} (n=7), MET^{AMP} (n=1) and Others (wildtype or not available; n=26), In addition, a comparison of *EGFR*$^{+}$ (n=3) and remaining subgroups is presented. C) Receiver operating characteristic (ROC) analysis of miRNA combi with the highest sensitivity (SE). D) ROC analysis of miRNA combi with the highest specificity (SP). E) Table displays the best miRNA combination panel with the highest SE and highest SP.

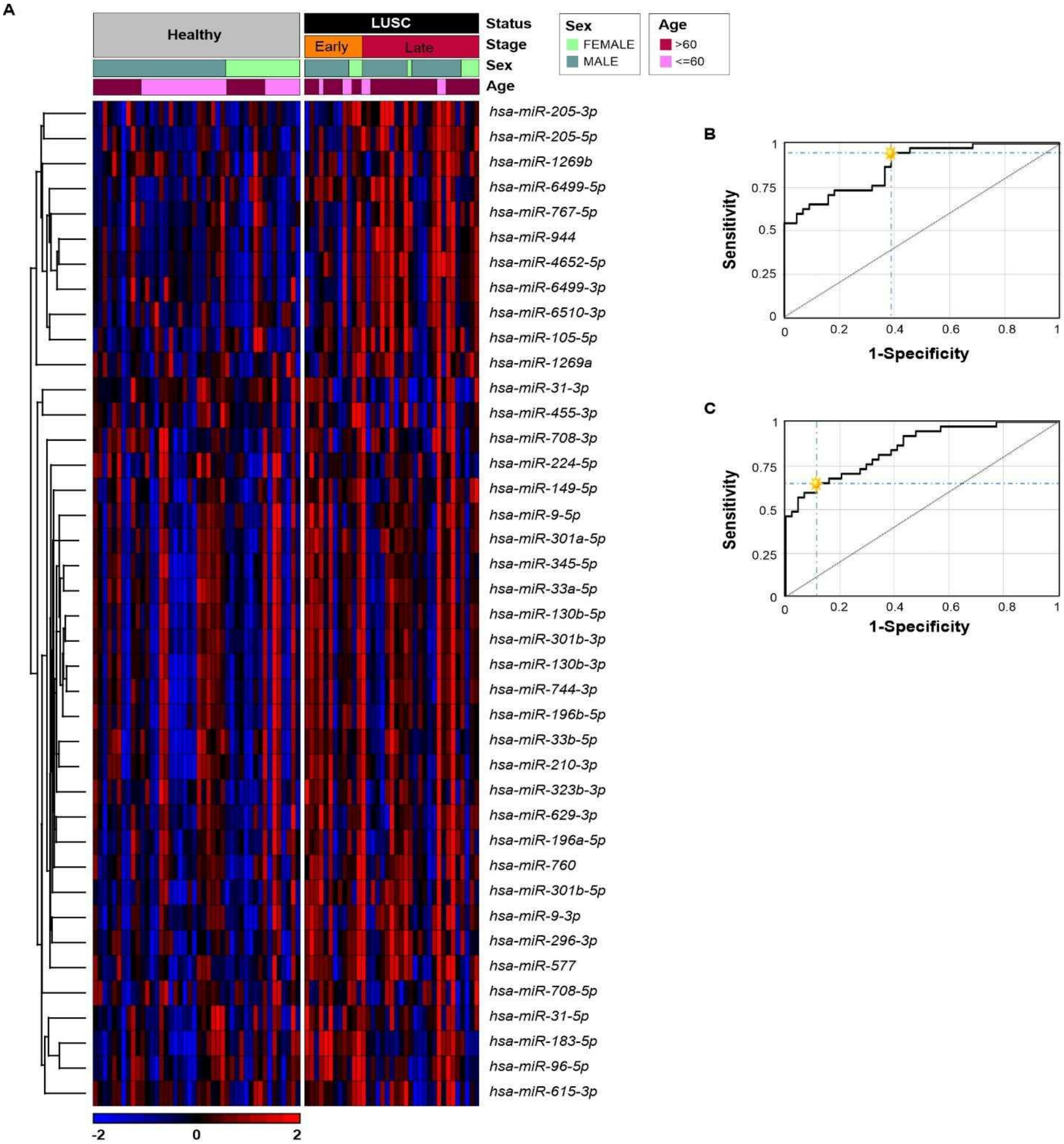

D

	Type	AUC	SE	SP	Cut-off	miRNAs Combination
LUSC Combi	Highest SE	0.87	0.95	0.61	0.28	hsa-miR-269-3p, hsa-miR-301b-5p, hsa-miR-4652-5p, hsa-miR-744-3p, hsa-miR-767-5p, hsa-miR-130b-3p, hsa-miR-205-3p
	Highest SP	0.85	0.65	0.89	0.56	hsa-miR-269-3p, hsa-miR-301b-5p, hsa-miR-130b-3p, hsa-miR-744-3p,hsa-miR-205-3p

Supplementary Figure 3. Comparison of plasma-derived miRNAs in LUSC patients and healthy individuals.

A) Heatmap shows the expression of all analyzed LUSC miRNAs (n=40) arranged according to clinical characteristics (stage, mutations, sex, and age). B) ROC analysis of miRNA combi with the highest sensitivity (SE). C) ROC analysis of miRNA combi with the highest specificity (SP). D) Table displays the best miRNA combination panel with the highest SE and highest SP.

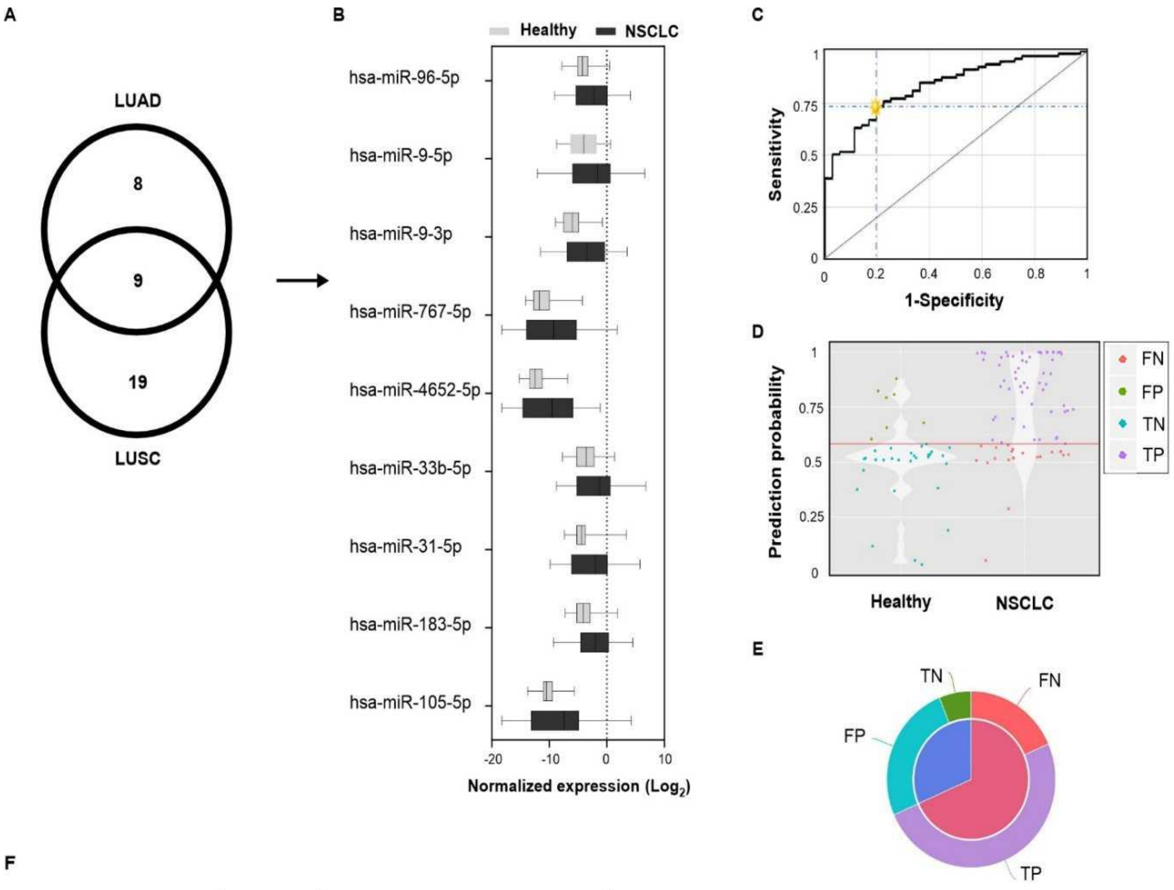

F

	Cohort	AUC	SE	SP	Cut-off	miRNAs Combination
Golden Combi	NSCLC	0.83	0.73	0.81	0.58	hsa-miR-183-5p, hsa-miR-31-5p, hsa-miR-33b-5p, hsa-miR-4652-5p, hsa-miR-9-3p, hsa-miR-9-5p, hsa-miR-96-5p
	Validation cohort	0.91	0.83	0.86	0.40	

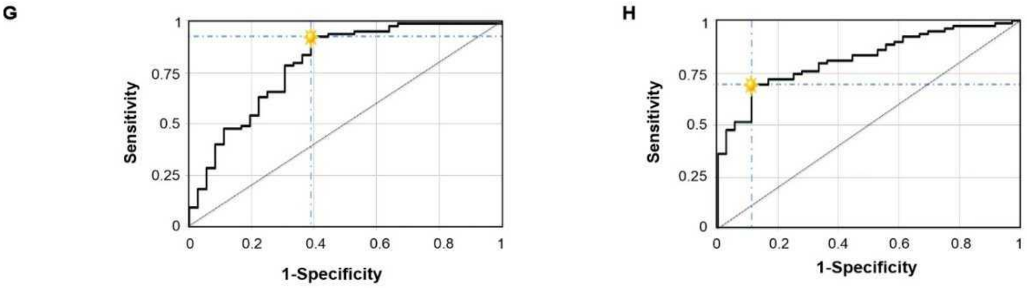

I

	Type	AUC	SE	SP	Cut-off	miRNAs Combination
NSCLC Combi	Highest SE	0.79	0.92	0.61	0.60	hsa-miR-183-5p, hsa-miR-31-5p,hsa-miR-4652-5p, hsa-miR-9-3p, hsa-miR-9-5p
	Highest SP	0.82	0.69	0.89	0.60	hsa-miR-105-5p, hsa-miR-31-5p, hsa-miR-33b-5p, hsa-miR-4652-5p, hsa-miR-9-3p, hsa-miR-9-5p, hsa-miR-96-5p

Supplementary Figure 4. Panel of differentially expressed overlapped miRNAs serve as non-invasive diagnostic biomarkers in NSCLC.

A) Venn diagram of overlapped DE miRNA (n= 9) in LUAD (n=17) and LUSC (n=28) subgroups. B) Boxplot shows the expression of nine shared miRNAs in NSCLC vs. healthy. Data is presented as means ±SD. C) ROC analysis reveals the best combination panel of DE miRNAs with the highest sensitivity (SE) and specificity (SP), and best area under the curve for the NSCLC. D) Violin plot shows the probability density of two compared sample groups (LUSC vs. healthy). E) Pie chart illustrates the percentages of false predictions (false positives, FPs; false negatives, FNs) and true predictions (true positives, TPs; true negatives, TNs). F) Table displays the best miRNA combination panel according to the highest AUC, SE, SP, and optimal cut-off in both NSCLC and validation cohorts as determined by the CombiRoc analysis. G) ROC analysis of miRNA combi with the highest sensitivity (SE). H) ROC analysis of miRNA combi with the highest specificity (SP). I) Table displays the best miRNA combination panel with the highest SE and highest SP.

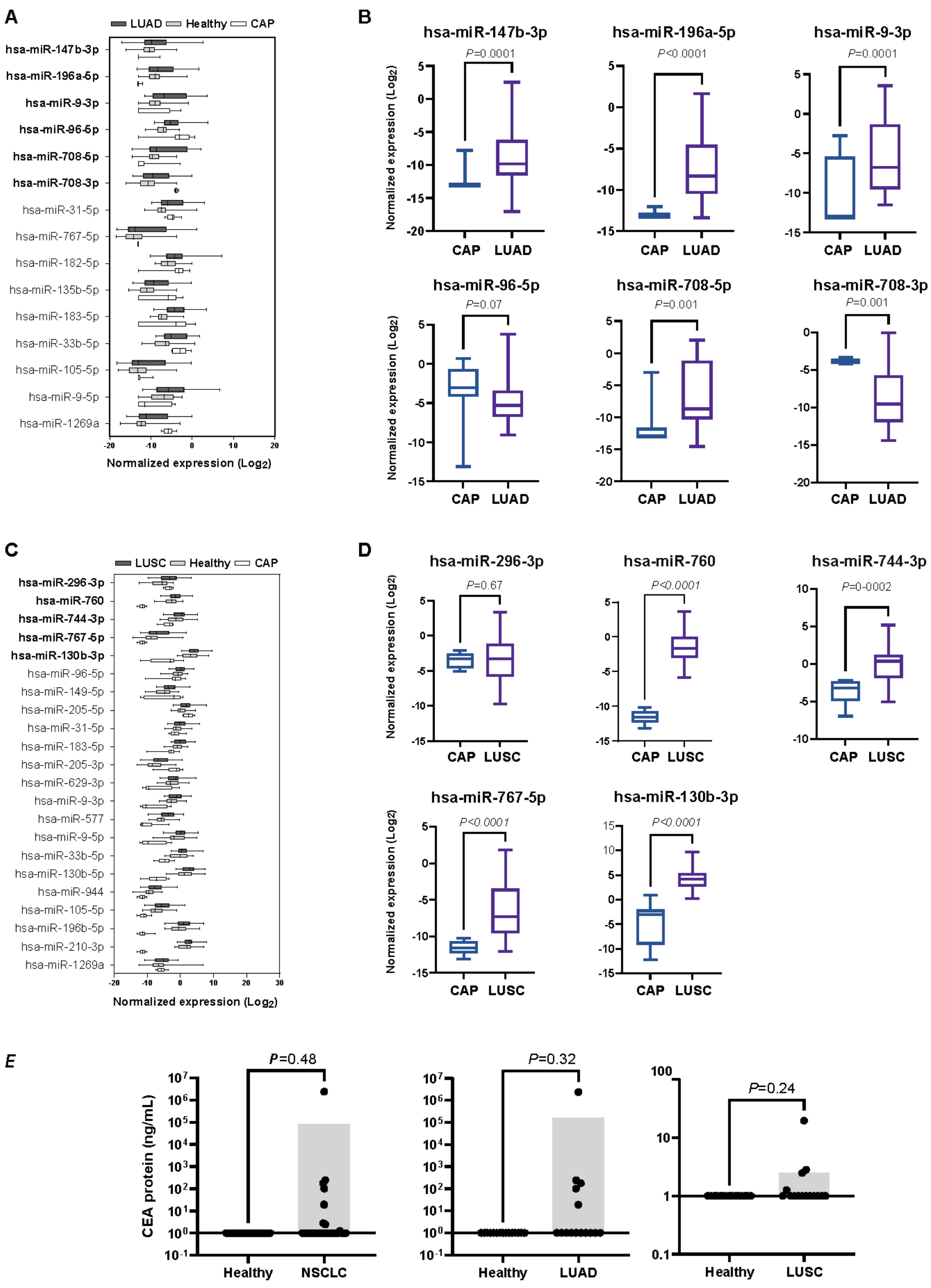

Supplementary Figure 5. Evaluation of differentially expressed miRNAs in NSCLC vs. CAP and comparison of CEA protein concentration.

A) Boxplot shows the expression profile of miRNAs in LUAD (n=41) vs. Community-Acquired Pneumonia (CAP; n=8) & healthy (n=36), LUAD diagnostic miRNA panel shown in bold. B) Boxplots representing the expression of LUAD golden combi miRNAs (n=6) compared to CAP. Data are displayed as means ± SD. C) Boxplot shows the expression of miRNAs in LUSC (n=37) vs. CAP (n=8) & healthy (n=44), LUSC diagnostic miRNA panel shown bold. D) Boxplots showing the expression of LUSC golden combi miRNAs (n=5)

comparison with CAP. Data are displayed as means ± SD. E) Carcinoembryonic antigen (CEA) protein quantification in the plasma cohort compiled of LUAD (n=15), LUSC (n=15), NSCLC (n=30) and healthy (n=15), assessed by ELISA.

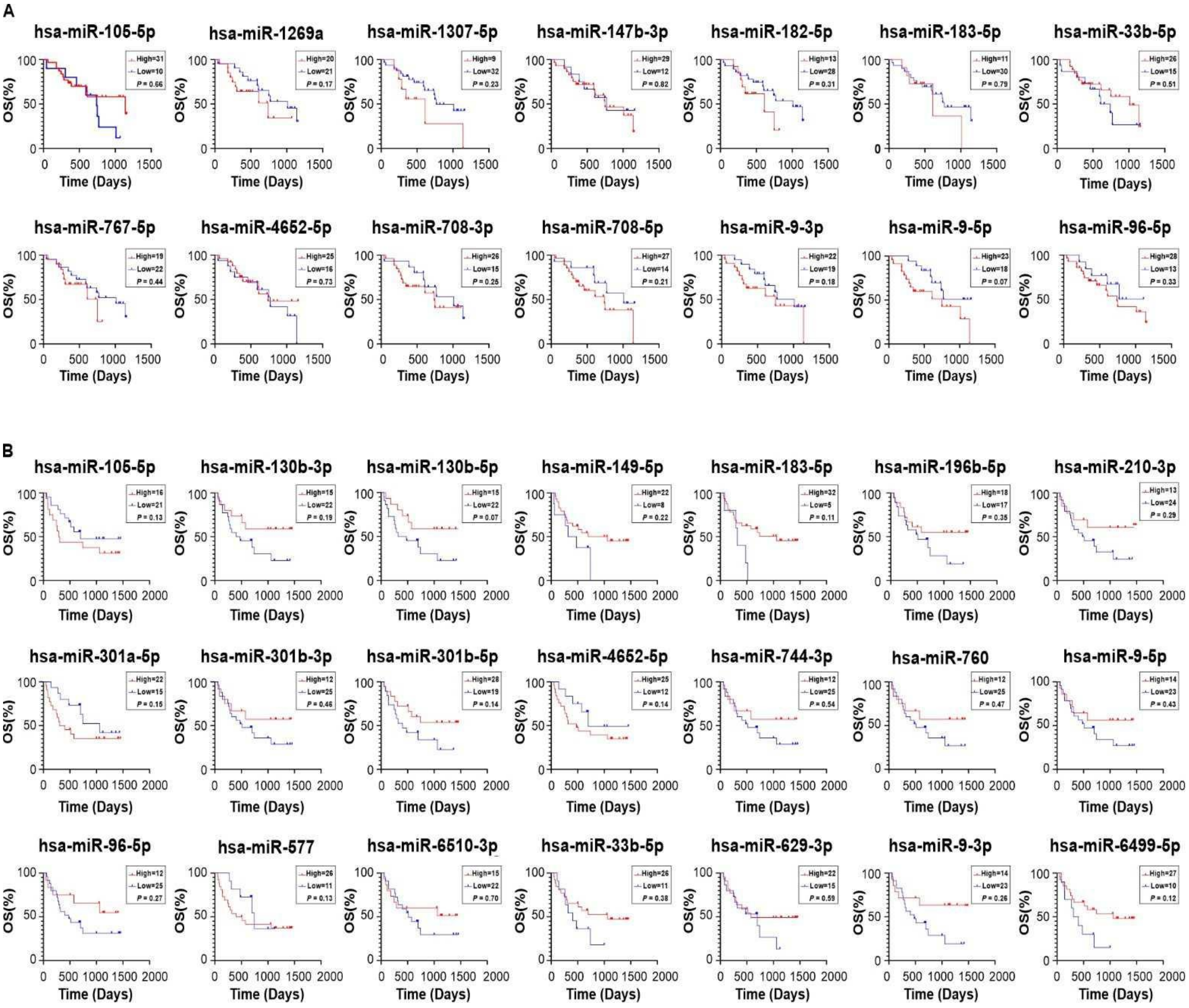

Supplementary Figure 6. Prognostic value of differentially expressed miRNAs as non-invasive biomarkers for NSCLC Subtypes.

A) Non-significant miRNA Kaplan-Meier overall survival (OS) plots in LUAD cohort (n=14, Log-rank test P<0.05). B) Non-significant miRNA OS plots in the LUSC cohort (n=21, Log-rank test P<0.05).

Supplementary Tables

Supplementary Table 1. Patient characteristics in NSCLC and healthy individuals.

**Variables**	**Study population (n=122)**		
	**Adenocarcinoma**	**Squamous cell carcinoma**	**Healthy**
	(*N*=41)	(*N*=37)	(*N*=44)
Age, years ±SD	66 ±9	69 ±10	60.23 ±7
Sex, *n* (%)			
Men	23 (56.0)	29 (78.3)	28 (63.6)
Women	18 (44.0)	8 (21.7)	16 (33.4)
Smoking per year			
Mean ±SD	40 ±24.91	50 ±16.9	7.88 ±13.50
Stage, n (%)			
I (IA + IB)	9 (21.9)	8 (21.6)	-
II (IIA + IIB)	2 (0.04)	4 (10.8)	-
III (IIIA + IIIB)	11 (26.8)	15 (40.5)	-
IV	19 (51.26)	10 (27.1)	-

SD, standard deviation.

Supplementary Table 2. Summary of the identified miRNA-based diagnostic, prognostic, and predictive biomarkers for NSCLC subtypes.

Cancer type	Diagnostic BM	Prognostic BM	Predictive BM
**LUAD**	Hsa-miR-147b-3p<br>Hsa-miR-196a-5p<br>Hsa-miR-4652-5p<br>Hsa-miR-708-3p<br>Hsa-miR-708-5p<br>Hsa-miR-9-3p<br>Hsa-miR-96-5p	Hsa-miR-135b-5p<br>Hsa-miR-196a-5p<br>Hsa-miR-31-5p	Hsa-miR-105-5p<br>Hsa-miR-1269a<br>Hsa-miR-135b-5p<br>Hsa-miR-182-5p<br>Hsa-miR-183-5p<br>Hsa-miR-196a-5p<br>Hsa-miR-33b-5p<br>Hsa-miR-767-5p<br>Hsa-miR-9-5p<br>Hsa-miR-96-5p
**LUSC**	Hsa-miR-301a-5p<br>Hsa-miR-301b-5p<br>Hsa-miR-6499-3p<br>Hsa-miR-744-3p<br>Hsa-miR-760<br>Hsa-miR-130b-3p<br>Hsa-miR-296-3p<br>Hsa-miR-4652-5p<br>Hsa-miR-767-5p	Hsa-miR-205-3p<br>Hsa-miR-205-5p<br>Hsa-miR-6499-3p<br>Hsa-miR-767-5p<br>Hsa-miR-944<br>Hsa-miR-31-5p<br>Hsa-miR-296-3p	-
**NSCLC**	Hsa-miR-183-5p<br>Hsa-miR-9-5p<br>Hsa-miR-9-3p<br>Hsa-miR-96-5p<br>Hsa-miR-4652-5p<br>Hsa-miR-33b-5p<br>Hsa-miR-31-5p	-	-

BM, biomarker.

Supplementary Table 3. Summary of the identified miRNA-based diagnostic biomarkers for NSCLC subtypes in different studies.

NSCLC subtype	miRNAs	Tissue BM	Circulating BM	PMID
LUAD	hsa-miR-147b-3p	Yes	No	37686123
	hsa-miR-196a-5p	No	Yes	31938164
	hsa-miR-708-3p	Yes	No	22573352
	hsa-miR-708-5p	Yes	No	22573352
	hsa-miR-9-3p	Yes	No	30761256
	hsa-miR-96-5p	Yes	No	36017148
LUSC	hsa-miR-301a-5p	Yes	No	27461635
	hsa-miR-296-3p	Yes	No	27186308, 31402954, 28751441
	hsa-miR-744-3p	Yes	No	34599436
	hsa-miR-760	Yes	No	35872931, 29869740
	hsa-miR-767-5p	Yes	Yes	32266038
	hsa-miR-130b-3p	Yes	No	33575477, 31061410
	hsa-miR-301b-5p	-	-	No study found
	hsa-miR-6499-3p	-	-	No study found
LUAD & LUSC	hsa-miR-4652-5p	Yes	No	35334070

BM, biomarker; PMID, PubMed ID.
